# Contribution of Nepal’s Free Delivery Care Policies in Improving Utilisation of Maternal Health Services

**DOI:** 10.15171/ijhpm.2018.01

**Published:** 2018-01-16

**Authors:** Hema Bhatt, Suresh Tiwari, Tim Ensor, Dhruba Raj Ghimire, Tania Gavidia

**Affiliations:** ^1^Oxford Policy Management/NHSSP, Kathmandu, Nepal.; ^2^Oxford Policy Management, Kathmandu, Nepal.; ^3^University of Leeds, Leeds, UK.; ^4^Volunteer VSO, Nepal.

**Keywords:** Free ِelivery Policies, Institutional Delivery, ANC Visits

## Abstract

**Background:** Nepal has made remarkable improvements in maternal health outcomes. The implementation of demand and supply side strategies have often been attributed with the observed increase in utilization of maternal healthcare services. In 2005, Free Delivery Care (FDC) policy was implemented under the name of Maternity Incentive Scheme (MIS), with the intention of reducing transport costs associated with giving birth in a health facility. In 2009, MIS was expanded to include free delivery services. The new expanded programme was named "Aama" programme, and further provided a cash incentive for attending four or more antenatal visits. This article analysed the influence of FDC policies, individual and community level factors in the utilisation of four antenatal care (4 ANC) visits and institutional deliveries in Nepal.

**Methods:** Demographic and health survey data from 1996, 2001, 2006 and 2011 were used and a multi-level analysis was employed to determine the effect of FDC policy intervention, individual and community level factors in utilisation of 4 ANC visits and institutional delivery services.

**Results:** Multivariate analysis suggests that FDC policy had the largest effect in the utilisation of 4 ANC visits and institutional delivery compared to individual and community factors. After the implementation of MIS in 2005, women were three times (adjusted odds ratio [AOR]=3.020, P<.001) more likely to attend 4 ANC visits than when there was no FDC policy. After the implementation of Aama programme in 2009, the likelihood of attending 4 ANC visits increased six-folds (AOR=6.006, P<.001) compared prior to the implementation of FDC policy. Similarly, institutional deliveries increased two times after the implementation of the MIS (AOR=2.117, P<.001) than when there was no FDC policy. The institutional deliveries increased five-folds (AOR=5.116, P<.001) after the implementation of Aama compared to no FDC policy.

**Conclusion:** Results from this study suggest that MIS and Aama policies have had a strong positive influence on the utilisation of 4 ANC visits and institutional deliveries in Nepal. Nevertheless, results also show that FDC policies may not be sufficient in raising demand for maternal health services without adequately considering the individual and community level factors.

## Background

Barriers to health service utilisation can be described as an interplay between demand-side and supply-side determinants. Demand-side determinants are factors influencing the ability to use health services at individual and community level. In contrast, supply-side determinants are derived from aspect inherent to the health system that either facilitate or hinder production of effective service and thereby influence service uptake by individual and community level.^1^ Until 2005, maternal health policies in Nepal focused on reducing the supply barriers. Despite the significant investments in addressing supply side factors, barriers in accessing services continue to persist.^2^ In order to improve access to and utilisation of maternal health services, Nepal started implementing demand side financing (DSF) intervention since 2005.^2^ DSF mechanism transfer purchasing power to specified groups for the purchase of defined goods or services.^3^ Vouchers for maternity services, vouchers for merit goods, conditional, un-conditional cash transfers and short term payments to offset the cost of assessing maternity services are some existing forms of DSF mechanism in maternal health.^3^ The ‘Maternity Incentive Scheme’ (MIS) was the first Free Delivery Care (FDC) policy aimed at improving institutional deliveries in Nepal.^2^ The MIS sought to reduce transportation costs associated with institutional deliveries, following the finding that reaching a facility accounted for almost two-thirds of the total cost of an institutional delivery.^4^ Through the MIS, all women giving birth in a government institutions or a listed private facilities, were eligible to receive NPR 1500 (US$15) if they lived in Mountain districts, NPR 1000 (US$10) in Hill districts and NPR 500 (US$5) in Tarai (flat land). The difference in the transport incentive was designed to address the high transportation cost in hard to reach areas, where travel was considered difficult and costly.^2^ The MIS was expanded to include ‘free deliveries’ in 2009.^5^ For Nepali women, this meant that in addition to receiving the transportation cash incentive, official user fees were removed for all types of deliveries from both public and accredited private facilities. The same year, Government of Nepal (GoN) introduced the “4 antenatal care (ANC) incentive program” to improve attendance to ANC. This meant all women completing four or more ANC in designated months (four, six, eight, and nine) were entitled to receive an additional NPR 400 (US$4) cash incentive. Together, these schemes are known as the FDC policy.^6^

Similar cash incentive programmes implemented in low and middle income countries have documented the positive effects of FDC policies to increasing uptake of ANC and institutional delivery services.^3,7^ An early evaluation of the Aama programme done by Powell-Jackson and colleagues in 2010 reported positive impact of Aama programme on the uptake of institutional delivery services.^8^ Birthing center accreditation framework and case based payment modality allowed government to purchase services from both public and private health facilities.^6^ As a result, by 2011 more public and private sector health facilities were willing to implement the Aama programme.^9^ In a recent study, Ensor and colleagues reported maternal health financing policies and its subsequent versions to be associated with the increase in institutional delivery in Nepal.^10^ More recent evidences also suggest of considerable improvements in the proportion of women attending 4 ANC visits (9% in 1995^11^ to 62% in 2016^12^) and institutional deliveries (9%^11^ to 57% in 2016^12^). These figures indicate that more women in Nepal are using maternal health services than ever before.

Despite the encouraging improvements from demand-side and supply side policies, a significant proportion of women fail to be benefitted from maternal health services. In the same study by Ensor and colleague suggest that maternal health financing policies are skewed towards areas and households that are geographically more accessible and wealthy.^10^ Quality of services, equity in service utilisation and value for money were key concerns raised by Murray and colleagues in their systematic analysis evaluating the effect of FDC policies in low- and middle-income countries.^3^ At the same time, a number of studies have identified key contextual factors such as background characteristics of the beneficiaries, level of health awareness, socio-cultural beliefs and service availability status to affect demand side interventions.^3,13^ This poses an important question around Nepal’s capacity to achieve universal healthcare coverage and ensuring leaving no one behind. In this context, this paper examines the contribution of FDC policies, individual and community factors in the utilisation of 4 ANC visits and institutional deliveries in Nepal.

### Data

This study analysed data from the Nepal Demographic and Health Survey (NDHS) 1996, 2001, 2006 and 2011. The Demographic and Health Survey is a two-stage stratified cluster sampling providing nationally representative estimates on key demographic and reproductive health indicators including the maternal health. In the first stage, primary sampling units (ward or a group of wards in rural and sub wards in urban) were selected from a sampling frame independently in each stratum. The sampling frame is a complete list of enumeration areas (EAs) created from the most recent population census. In the second stage, a fixed number of households were selected from the household list in each of the selected EAs, and all household members in a certain age group (eg, all women age 15-49 and all men age 15-59).Details of the sampling methodology can be obtained elsewhere.^14^ The NDHS datasets are publicly available upon request through Measure DHS website.^15^ The NDHS 2001, 2006, and 2011 captured information from five year preceding the survey,^16-18^ while NDHS 1996 captured information from three year preceding survey.^11^ To analyze maternal healthcare utilisation, a maternal dataset was created by merging relevant information from two data sets: household and birth from each round of survey. NDHS 1996, 2001, 2006 and 2011 reported information on 3845, 4731, 4182 and 4079 last born children. The analysis was restricted to only the last born children. A total of 16 837 births and related information have been analysed in this study.^11,16-18^ Appropriate weights were calculated using the methods described in DHS handbook.^14^ These weights were applied during the analysis.

## Methods

### Conceptual Framework

Andersen’s Behavioral model was used to conceptualise the determinants of 4 ANC visits and institutional delivery. Andersen’s model and its subsequent adaptations have been widely applied by studies investigating healthcare utilisation.^19-21^ According to the model, health service utilisation is determined by characteristics of the individual, the community in which the individual lives and the health system characteristics.^19,20^ Health-system factors refer to policies and programme intervention, resources and service delivery structure that influences on making health services accessible, available, acceptable and affordable to the people. For this paper, health system factors were measured only in terms of implementation of FDC policies. Individual factors included the demographic, and the socio-economic characteristics of a person that enables him/her to make use of health services. Individual factors analysed in the study were age, parity, education, caste/ethnicity and household wealth. Community factors include characteristics of a community in which the individual lives such as place of residence (rural/urban), geographic location (referred to as ecological belt and denoted by mountain, hill and tarai [flat land]), and administrative division (referred to as development region and denoted by eastern, central, western, mid-western and far-western region). Similarly, other forms of community variables investigated in this paper were community concentration of the rich households and the high caste households. Many studies argue that individual level characteristics to be the most important factors influencing health service utilisation as both the community and health-systems factors were measured within the context of individual healthcare utilisation.^20,21^

### Statistical Analysis

This paper investigated the determinants of two outcome variables, 4 ANC visits and utilisation of institutional delivery. In order to examine the characteristics of the sample, a descriptive analysis was conducted for all variables. The association between 4 ANC visits, utilisation of institutional delivery and independent variables were examined first through unadjusted odds ratios (ORs) denoted as model I. Significant FDC policy, individual and community variables from model I were then included in the multiple logistic regression model II, and model III respectively. In the final model IV significant FDC policy, individual and community variables were added to generate adjusted ORs. For the multiple logistic regression analysis, a hierarchical modeling strategy was used.^22^ *P* value <.05 was considered as statistically significant. The analysis was performed using the survey command in Stata 13 -svy-, which allows for the complex sample design of the survey.^23^

Prior to multiple regression analysis, independent variables were tested for co-linearity between independent variables.^22,24^ Co-linearity was said to exist between variables when r = 0.71. Parity was found to be negatively correlated with all the independent variables. A fairly strong correlation was observed in household wealth and concentration of the rich households (r = 0.63). Correlation was found in concentration of the high caste and caste/ethnicity (r = 0.55). As the r value did not cross threshold limit, all variables were included in the analysis.

### Measures

#### Dependent Variables

Two indicators of maternal healthcare utilisation were investigated. The first, was whether or not a woman had at least 4 ANC visits during her most recent pregnancy in the five year period preceding the survey. The second was whether or not a woman had given birth in an institution, either public or private health facilities, hospitals or health posts.

#### Independent Variables

Common determinants of 4 ANC visits and institutional delivery were grouped into three broader categories: FDC policy, individual and community level factor.^21-28^

Demand side financing policy variable was created from the date of birth of the last born children segregated into three groups (*i*). ‘No FDC policy covering the period between 1994-2004,’ (*ii*). ‘MIS’ covering 2005-2008 period, and (*iii*). ‘Aama’ covering period between 2009-2011. Individual level factors included were age at birth, parity, husband’s education, woman’s education, caste/ethnicity, household wealth index. Age was divided into four categories including ‘15-19,’ ‘20-29,’ ‘30–39,’ and ‘≥40’ years. Parity was grouped into three categories ‘1,’ ‘2–3,’ and ‘≥4.^23,24^ Women’s education indicates the highest levels of education completed by the respondent and were grouped into ‘no education,’ ‘less than secondary education,’ ‘secondary education or high.’^21^ Caste/ethnicity in this study was categorised based on previously published NDHS related studies (*i*) Advantaged caste groups included members from Brahmin, Chhetri, and Newar groups (ie, upper cast community groups) (*ii*) Disadvantaged Janjati/Trible included disadvantaged indigenous Janjati and Muslims and (*iii*) Disadvantaged Dalit (Scheduled cast/Sub Cast/low cast) included Dalit and Tarai madhesi (ie, local Community of Plain).^22,29^ The NDHS wealth index was used as a measure of economic status. The NDHS wealth index was calculated using principle component analysis of more than 40 household assets, grouping households into wealth quintiles.^30,31^ Due to the similarity between wealth quintiles, Agho and colleagues suggested to re-categorise these quintiles into (*i*) Lowest 40% as poor, (*ii*) middle 40% as middle, and (*iii*) upper 20% as rich.^32^

Community-level factors include place of residence measured as rural and urban. Similarly, location of residence by ecological belt was based on the altitude division of the country into belt’s namely (*i*) ‘mountain,’ (*ii*) hill, (*iii*) tarai (flat land). Location of residence by development region was based on administrative divisions of the country into five vertical sections namely (*i*) eastern development region, (*ii*) central development region, (*iii*) western development region, (*iv*) mid-western development region, and (*v*) far-western development region.^22,24^ Some community variables were constructed by aggregating the individual characteristics of respondents to PSU.^21^ The PSU was considered as the community level of analysis in this paper. Concentration of the rich was calculated by calculating the%age of households in each PSU that fall in the richest 20%. PSU were then grouped into low, medium and high concentration of the rich.^21^ Concentration of the upper caste reflects the%age of households in the each PSU that fall in the advantaged caste/ethnicity group. PSU were then grouped into low, medium and high concentration of advantaged caste/ethnicity.^21^

## Results

### Sample Characteristics

Table 1 presents general characteristics of 16 837 women of reproductive age who participated in NDHS survey 1996, 2001, 2006 and 2011. Characteristics were disaggregated by FDC policy intervention year (no FDC policy, MIS, and Aama) to provide a clear picture of the uptake of 4 ANC visits and delivery services over time. Fifteen percent of women had 4 ANC visits for their most recent pregnancy in 1994-2004, which reached to 37% in 2005-2008 and 54% in 2009-2011. Similarly, 19% women reported of giving birth in a health facility between 1994-2004, 24% in 2005-2008 and 42% in 2009-2011. The majority women were between the ages of 15 to 29 years at the time of their last child birth. Most women already had either two or three children. Over the policy implementation year and before, majority women have no education; however by 2009-2011, 38% women were found to have secondary or high education. One-third of women belonged to disadvantaged Dalit caste. Almost half of the women belonged to low income household. More than two third women were from rural locality. Majority were from tarai (flat land). Less than one-third women were from western development region. More than 75% belonged to communities with low concentration of the rich households and almost 50% were from communities with low concentration of the high caste households.

**Table 1:** General Characteristics of Women Aged 15-49 Years From NDHS 1996, 2001, 2006 and 2011 Disaggregated by FDC Policy Intervention Year (N = 16 837)

**Variables**	**1994-2004 (No FDC Policy)** **No. (%)**	**2005-2008 (MIS) ** **No. (%)**	**2009-2011 (Aama) ** **No. (%)**
**Dependent Variable**			
4 ANC			
No	9047 (84.9)	2119 (62.6)	1267 (45.3)
Yes	1608 (15.1)	1268 (37.4)	1528 (54.7)
Institutional Delivery			
No	9512 (89.3)	2546 (75.2)	1600 (57.3)
Yes	1143 (10.7)	841 (24.8)	1195 (42.7)
**Individual Level Variable**			
Age at birth				
15-19	803 (7.3)	290 (8.6)	291 (10.4)
20-29	6183 (58.1)	2132 (63.0)	1875 (67.1)
30-39	2984 (28.1)	814 (24.0)	548 (19.6)
≥40	685 (6.5)	151 (4.4)	81 (2.9)
Parity			
Birth order 1	2252 (21.1)	946 (27.9)	941 (33.7)
Birth order 2-3	4287 (40.3)	1488 (43.9)	1244 (44.5)
Birth order 4+	4116 (38.6)	953 (28.2)	610 (21.8)
Women’s education			
No education	7742 (72.7)	1782 (52.7)	1172 (41.9)
Less than secondary education	1449 (13.6)	658 (19.4)	556 (19.9)
Secondary or high	1464 (13.7)	947 (27.9)	1067 (38.2)
Caste/ethnic group			
Disadvantage Dalit: Dalit and tarai Madhesi	2888 (27.2)	844 (25.0)	703 (25.2)
Disadvantage Janjati: Janjati, Muslim and Others	3934 (36.9)	1185 (34.9)	980 (35.1)
Advantage: Brahman, Chhetri and Newar	3833 (35.9)	1358 (40.1)	1112 (39.7)
Household income group			
Low income: poorest 40%	4854 (45.5)	1636 (48.3)	1386 (49.6)
Middle income: middle 40%	4072 (38.2)	1197 (35.3)	987 (35.3)
High income: richest 20%	1729 (16.3)	554 (16.4)	422 (15.1)
**Community Level Variable**			
Ecological belt			
Mountain	1518 (14.3)	543 (16.1)	537 (19.2)
Hill	4210 (39.5)	1329 (39.2)	1121 (40.1)
Tarai	4927 (46.2)	1515 (44.7)	1137 (40.7)
Place of residence			
Rural	9316 (87.4)	2622 (77.4)	2199 (78.7)
Urban	1339 (12.6)	765 (22.6)	596 (21.3)
Development region			
Eastern	2267 (21.3)	786 (23.2)	640 (22.9)
Central	2949 (27.7)	761 (22.6)	585 (20.9)
Western	1914 (17.9)	579 (17.1)	440 (15.8)
Mid-western	1688 (15.9)	633 (18.6)	630 (22.6)
Far-western	1837 (17.2)	628 (18.5)	500 (17.8)
Concentration of the rich households			
Low	8017 (75.2)	2486 (73.4)	2076 (74.3)
Medium	1736 (16.3)	672 (19.8)	559 (20.0)
High	902 (8.5)	229 (6.8)	160 (5.7)
Concentration of the high caste households			
Low	6130 (57.5)	1753 (51.8)	1411 (50.5)
Medium	2964 (27.8)	1079 (31.9)	1004 (35.9)
High	1561 (14.7)	555 (16.3)	380 (13.6)

Abbreviations: FDC, Free Delivery Care; ANC, antenatal care; MIS, Maternity Incentive Scheme; NDHS, Nepal Demographic and Health Survey.

### Trend in 4 Antenatal Care Visits and Utilisation of Delivery Care

Figure suggests an increasing trend in 4 ANC visits and institutional deliveries over the period of 18 years. Between 1994 and 2011, women visiting 4 ANC has increased from 9.2% to 54.3%. A similar growth was also noted for institutional delivery from 6.6% in 1994 to 45.6% in 2011. More than 95% of 4 ANC visits and institutional deliveries were conducted in public facilities.^11,16-18^

**Figure:**
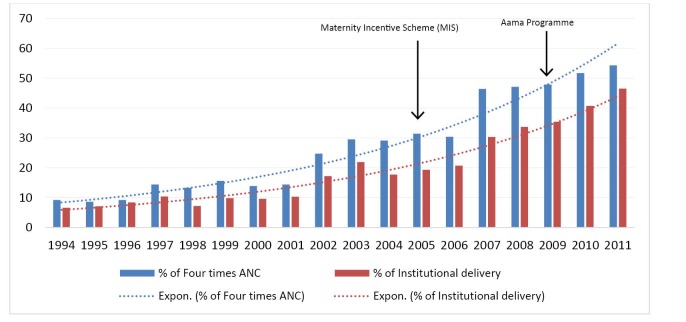
Trend in 4 ANC Visits, Utilisation of Institutional Delivery Between 1994-2011. Abbreviation: ANC, antenatal care.
Source: NDHS 1996, 2001, 2006, 2011.

### Factors Associated With 4 Antenatal Care Visits

Multivariate analysis was used to identify factors most strongly linked with 4 ANC visits over the years and to ascertain the relative influence of FDC policy, individual and community level factors. Based on the conceptual framework, univariate analysis of the FDC policy, individual and community variable was presented in first column (referred as model I). Model II, second column presents analysis after adjusting for all individual variables. Model III, third column presents analysis after adjusting for community variables. The final model, model IV presents analysis after adjusting for all FDC policy, individual and community variables (see Table 2).

**Table 2:** FDC Policy, Individual and Community Factors Contributing 4 ANC Visits

**Variables**	**Model I**	**Model II**	**Model III**	**Model IV**
	**OR (95% CI)**	**OR (95% CI)**	**OR (95% CI)**	**OR (95% CI)**
FDC Policy			
(ref = No FDC policy, 1994-2004)
MIS 2005-2008	3.319 (2.829, 3.918)^a^			3.020 (2.582, 3.531)^a^
Aama 2009-2011	5.999 (4.769, 7.547)^a^			6.006 (4.824, 7.477)^a^
**Individual Level Variables**				
Age at birth (ref = more than 40)				
15-19	5.426 (3.854, 7.638)^a^	0.834 (0.567, 1.225)		0.825 (0.548, 1.243)
20-29	5.719 (4.155, 7.871)^a^	1.277 (0.900, 1.812)		1.288 (0.899, 1.843)
30-39	3.109 (2.258, 4.282)^a^	1.588 (1.154, 2.187)^b^		1.531 (1.096, 2.140)^c^
Parity (ref = 1)				
2-3	0.538 (0.489, 0.592)^a^	0.626 (0.554, 0.706)^a^		0.615 (0.542, 0.697)^a^
4 or more	0.152 (0.131, 0.177)^a^	0.291 (0.237, 0.357)^a^		0.318 (0.260, 0.388)^a^
Women’s education (ref = no education)				
Primary	3.537 (3.144, 4.061)^a^	2.573 (2.249, 2.943)^a^		2.029 (1.769, 2.327)^a^
Secondary and above	11.419 (9.727, 13.405)^a^	5.178 (4.442, 6.036)^a^		3.227 (2.798, 3.723)^a^
Caste/ethnic group (ref = Disadvantage Dalit: Dalit and tarai Madhesi)				
Disadvantage Janjati: Janjati, Muslim and Others	1.279 (1.049, 1.565)^c^	0.992 (0.804, 1.224)		1.034 (0.820, 1.304)
Advantage: Brahman, Chhetri and Newar	2.741 (2.266, 3.316)^a^	1.372 (1.158, 1.625)^a^		1.547 (1.277, 1.875)^a^
Household income group (ref = Low income: 40%)				
Middle income: middle 40%	2.308 (1.977, 2.694)^a^	1.658 (1.431, 1.921)^a^		1.734 (1.476, 2.037)^a^
High income: richest 20%	7.715 (6.342, 9.385)^a^	2.922 (2.406, 3.548)^a^		2.734 (2.185, 3.421)^a^
**Community Variable**				
Ecological belt (ref = mountain)				
Hill	1.703 (1.238, 2.342)^a^		1.325 (0.972, 1.806)	1.132 (0.854, 1.500)
Tarai	1.672 (1.258, 2.223)^a^		1.575 (1.146, 2.165)^b^	1.315 (0.971, 1.781)
Place of residence (ref = rural)				
Urban	4.003 (3.237, 4.949)^a^		1.638 (1.266, 2.118)^a^	1.296 (1.029, 1.632)^c^
Development region (ref = Far-western)				
Eastern	1.085 (0.754, 1.561)		1.040 (0.656, 1.649)	
Central	0.960 (0.661, 1.393)		0.852 (0.540, 1.344)	
Western	1.110 (0.766, 1.609)		1.012 (0.634, 1.617)	
Mid-western	0.699 (0.462, 1.059)		0.738 (0.462, 1.179)	
Concentration of rich (ref = low)				
Medium	3.151 (2.556, 3.961)^a^		2.418 (1.909, 3.064)^a^	1.332 (1.073, 1.654)^b^
High	6.412 (4.841, 8.493)^a^		3.924 (2.801, 5.496)^a^	1.908 (1.350, 2.697)^a^
Concentration of high caste (ref = low)				
Medium	2.140 (1.696, 2.702)^a^		1.831 (1.460, 2.296)^a^	1.198 (0.987, 1.456)
High	1.870 (1.372, 2.548)^a^		1.872 (1.385, 2.530)^a^	1.264 (0.993, 1.609)

Abbreviations: FDC, Free Delivery Care; ANC, antenatal care; MIS, Maternity Incentive Scheme. Note:^a^ ≤.001, ^b^ ≤.01 and ^c^ ≤.05.

Result of the multivariate analysis suggests that FDC policy has the largest effect of all variables included in the model. Women who became pregnant after the implementation of MIS, were three times more likely (OR = 3.319, *P* < .001) to have attended 4 ANC visits compared to women who were pregnant when there was no FDC policy in between 1994-2004. Women who were pregnant after the implementation of Aama in 2009, were almost six times more likely (OR = 5.999, *P* < .001) to have attended 4 ANC. After adjusting for FDC policy, individual and community level factors, the likelihood of women attending 4 ANC visits did not change much from their crude values. After the implementation of the MIS, women remained three times more likely to attend 4 ANC compared to women who were pregnant when there was no incentive scheme (adjusted OR [AOR] = 3.020, *P* < .001). Similarly, women remained six times (AOR = 6.006, *P* < .001) more likely to have attended 4 ANC after the implementation of Aama.

All individual variables from model I remained significant in the final model. In model IV, household wealth, caste/ethnicity (advantage caste) and age at birth (30-39 years old) were found to have significantly increased the odds of attending 4 ANC. However, women’s education was found to be dominant individual variable with the largest effect size. The odds of attending 4 ANC visits increased with women’s education. Women with primary education were two times (AOR = 2.029, *P* < .001) more likely to have attended 4 ANC visits where as those who have secondary or high education were three times (AOR = 3.227, *P* < .001) more likely to have attended 4 ANC visits compared to women with no education.

The odds of attending 4 ANC visits also increased with increasing household wealth. Compared to women from poor households, the odds for attending 4 ANC was 1.7 times (AOR = 1.734, *P* < .001) for women belonging to medium levels of household wealth and almost three folds (AOR = 2.734, *P* < .001) for women belonging to high levels of household wealth. Bhramin, Chettri and Newar women were 1.5 times (AOR = 1.547, *P* < .001) more likely to have attended 4 ANC compared to Dalit and tarai Madhesi groups. Women aged between 30-39 years were 1.5 times (AOR = 1.531, *P* < .01) more likely to have attended 4 ANC visits compared to older counterparts. Parity has negative effect on 4 ANC visits.

Place of residence and community concentration of rich households were the only community variables from model III that remained significant in the final model, however with a reduced effect size. After adjustment, ecological belt, development region and community concentration of the high caste households were no longer found to be significant. Community concentration of the rich households was found to be the dominant community variable with the largest effect size. The odds of attending 4 ANC visits was two times (AOR = 1.908, *P* < .001) for women residing in high concentration of the rich households compared to women living in communities with low concentration of the rich households.

### Factors Associated With Institutional Delivery

Table 3 presents the univariate and multivariate analysis of the FDC policy, individual and community variables on institutional delivery. A similar approach to analysis was taken as that of the 4 ANC visits.

**Table 3:** FDC Policy, Individual and Community Factors Contributing to the Utilisation of Institutional Delivery

**Variable**	**Model I**	**Model II**	**Model III**	**Model IV**
	**OR (95% CI)**	**OR (95% CI)**	**OR (95% CI)**	**OR (95% CI)**
FDC policy				
(ref = No FDC policy, 1994-2005)
MIS (2005-2008)	2.782 (2.340, 3.307)^c^			2.117 (1.764, 2.540)^c^
Aama (2009-2011)	5.916 (4.722, 7.411)^c^			5.116 (4.118, 6.357)^c^
**Individual Level Variables**				
Age at birth (ref = more than 40)				
15-19	3.287 (2.362, 4.573)^c^	0.373 (0.245, 0.568)^c^		0.348 (0.226, 0.534)^c^
20-29	2.756 (1.995, 3.807)^c^	0.426 (0.289, 0.628)^c^		0.408 (0.273, 0.608)^c^
30-39	1.590 (1.128, 2.240)^c^	0.597 (0.417, 0.855)^b^		0.569 (0.403, 0.831)^b^
Parity (ref = 1)				
2-3	0.391 (0.351, 0.435)^c^	0.462 (0.399, 0.535)^c^		0.422 (0.361, 0.493)^c^
4 or more	0.126 (0.106, 0.149)^c^	0.270 (0.216, 0.337)^c^		0.272 (0.217, 0.341)^c^
Visit of 4 or more ANC (ref = no)				
Yes	11.008 (9.368, 12.935)^c^	5.130 (4.440, 5.928)^c^		3.585 (3.105, 4.141)^c^
Women’s education (ref = no education)				
Primary	3.041 2.571, 3.596)^c^	1.587 (1.317, 1.912)^c^		1.360 (1.113, 1.661)^b^
Secondary and above	11.843 (9.962, 14.080)^c^	2.931 (2.416, 3.555)^c^		2.211 (1.803, 2.711)^c^
Caste/Ethnic group (ref = Disadvantage Dalit: Dalit and tarai Madhesi)				
Disadvantage Janjati: Janjati, Muslim and Others	1.092 (0.871,1.368)	0.749 (0.593, 0.947)^a^		0.818 (0.657, 1.017)
Advantage: Brahman, Chhetri and Newar	2.319 (1.879, 2.862)^c^	0.832 (0.683, 1.194)		1.084 (0.855, 1.374)
Household income group (ref = Low income: 40%)				
Middle income: middle 40%	2.780 (2.350, 3.288)^c^	1.744 (1.485, 2.049)^c^		1.651 (1.399, 1.950)^c^
High income: richest 20%	13.225 (10.706, 16.338)^c^	4.675 (3.807, 5.742)^c^		2.951 (2.324, 3.747)^c^
**Community Variable**				
Ecological belt (ref = mountain)				
Hill	2.666 (1.942, 3.660)^c^		1.665 (1.223, 2.267)^b^	1.552 (1.163, 2.070)^b^
Tarai	2.816 (2.089, 3.796)^c^		1.274 (1.520, 2.900)^c^	1.720 (1.279, 2.313)^c^
Place of residence (ref = rural)				
Urban	7.127 (5.669, 8.959)^c^		2.459 (1.911, 3.163)^c^	2.126 (1.752, 2.579)^c^
Development region (ref = Far western)				
Eastern	1.779 (1.208, 2.619)^c^		1.558 (1.084, 2.239)^a^	1.182 (0.852, 1.634)
Central	2.057 (1.426, 2.967)^c^		1.671 (1.186, 2.353)^b^	1.588 (1.155, 2.148)^b^
Western	1.616 (1.119, 2.334)^c^		1.363 (0.937, 1.983)	0.995 (0.724, 1.365)
Mid-western	1.094 (0.720, 1.662)		1.229 (0.825, 1.831)	1.293 (0.930, 1.799)
Concentration of rich (ref = low)				
Medium	4.307 (3.360, 5.521)^c^		3.051 (2.348, 3.965)^c^	1.652 (1.318, 2.069)^c^
High	11.931 (9.031, 15.763)^c^		5.262 (3.819, 7.252)^c^	2.434 (1.831, 3.236)^c^
Concentration of high caste (ref = low)				
Medium	2.127 (1.660, 2.726)^c^		1.728 (1.388, 2.152)^c^	1.027 (0.834, 1.263)
High	1.724 (1.215, 2.446)^b^		1.754 (1.288, 2.388)^c^	1.033 (0.791, 1.349)

Abbreviations: FDC, Free Delivery Care; ANC, antenatal care; MIS, Maternity Incentive Scheme. Note:^a^ ≤.001, ^b^ ≤.01 and ^c^ ≤.05.

Results of the multivariate analysis suggests that FDC policy has the largest effect of all variables included in the model. Women who gave birth after the implementation of MIS, were three times (OR = 2.782, *P* < .001) more likely to have institutional deliveries compared to women who gave birth when there was no FDC policy during 1994-2004. Women giving birth after the implementation of Aama in 2009, were almost six times more likely (OR = 5.916, *P* < .001) to have institutional deliveries. After adjusting for FDC policy, individual and community level factors, the likelihood of women giving birth in an institution changed slightly from the crude values. After the implementation of MIS, women were two times more likely to have attended institutional delivery compared to women who delivered when there was no incentive scheme (AOR = 2.017, *P* < .001). Similarly, women delivering after the implementation of free delivery policy were five times (AOR = 5.116, *P* < .001) more likely to have attended institutional delivery.

Except for caste/ethnicity all the individual variables from model I remained significant in the final model however with reduced effect size. After adjusting, household wealth and women’s education were found to have significantly increased the odds of institutional delivery. However, women’s attendance of 4 ANC was found to be dominant individual variable with the largest effect size. Women completing 4 ANC visits were almost four times more likely (AOR = 3.585, *P* < .001) to attend institutional delivery compared to women with no ANC. The odds of attending institutional delivery increased with increasing household wealth and education. Compared to women belonging to poor households, women belonging to medium levels of household wealth were 1.6 times (AOR = 1.651, *P* < .001) more likely to attend institutional delivery and those from high levels of household wealth were three times (AOR = 2.951, *P* < .001) more likely to attend institutional delivery. Women who were secondary or high educated were twice more likely (AOR = 2.211, *P* < .001) to utilise institutional delivery compared to no education. Caste had no significant effect in the utilisation of institutional delivery. Parity and age at birth both had negative effect on the utilisation of institutional delivery.

Except for community concentration of the high caste households, all variables from model II were still found to be significant in the final model however with the reduced effect size. After adjustment, place of residence, ecological belt and development region (women living in central region) were found to be significantly increasing the odds of attending institutional delivery. However, community concentration of the rich households was found to be dominant community variable with the largest effect size. Women residing in communities with medium concentration of the rich households and high concentration of the rich households were 1.6 times (AOR = 1.652, *P* < .001) to 2.4 times (AOR = 2.434, *P* < .001) more likely to have utilised institutional delivery compared to women living in communities with low concentration of the rich households. Women residing in urban areas were two times (AOR = 2.126, *P* < .001) more likely to have utilised institutional delivery compared to rural counterparts. The odds of utilising institutional delivery in hilly areas was 1.5 folds (AOR = 1.552, *P* < .01) where as in tarai, it was 1.7 folds (AOR = 1.720, *P* < .001) compared to mountain region. Women from central region were 1.5 times (AOR = 1.588, *P* < .005) more likely to have institutional delivery compared to far western region.

## Discussion

This paper suggests that utilisation of 4 ANC visits and institutional delivery in Nepal has increased over the last two decades. The findings demonstrate influence of FDC policy, individual and community level factors in the utilisation of both 4 ANC visits and institutional delivery. Compared to individual and community factors, FDC policy in the form of MIS and Aama had the greatest influence on the utilisation of 4 ANC and institutional delivery. These findings are well supported by an early evaluation of Aama programme, which suggested that with the introduction of FDC, the utilisation of public sector maternity services have increased.^33^ The recent studies also reported that MIS and its subsequent forms were found to have a positive effect on utilisation of maternal health services in Nepal.^8,10^

Earlier literature on maternal health financing suggests that improvement of supply side factors are essential for the success of FDC policy interventions.^1,3,35^ It is important to note that the GoN has increased its investments in supply side functions since 2005.^2,34^ Some of the most notable supply side interventions that were aimed at improving utilisation include the construction and expansion of birthing centers (BCs), strengthening emergency obstetric care, production of auxiliary nurse midwives and training of skilled birth attendant.^34^ In this paper, it was not possible to adequately capture the effect of supply-side interventions because DHS survey does not collect supply side information. However, it was important to note that a combination of demand and supply side strategies are important to bring the positive impact in the access to and utilisation of maternal health services.^1,3,35-37^ Administrative data suggests that the birthing center accreditation framework included in Aama policy has been instrumental in rapid expansion of birthing facilities and securing financial resources from the local government.^34^ Monitoring data from Ministry of Health suggests that local government has provided NPR 300 million (US$3 million) in building birthing centers and paying salary of additional nursing staffs to run BCs for 24x7.^34^ The number of birthing centers increased from 541 in 2007 to 894 in 2010 and to 2001 by 2016. Specifically, the number of lower level health facilities (sub/health post) providing delivery care significantly increased from a few hundreds to more than thousands. Together, the investments made in the healthcare system over the last 17 years, is believed to have brought services closer to the poor and women residing in hard to reach areas.^34^

The overarching goal of FDC policy intervention is to benefit those who are in need and ensure equity in utilisation of maternal health services.^6^ The universal nature of MIS and Aama policy intervention was aimed at making services equally available to all pregnant women irrespective of their age, education, caste, income, and geographical location.^6^ Evidence from this study indicates that not all individual and community characteristics are equally influenced by MIS and Aama policy. At the same time, Gopalan and colleagues including others have identified that the background characteristics of beneficiaries, level of health awareness, socio-cultural beliefs and service availability to affect the demand side interventions.^3,13^ Ensor and colleagues in their a recent publication confirm that the MIS and Aama policies skewed towards areas and households that were wealthier and geographically more accessible in Nepal.^10^ Similar to other studies^8,21,24-28,33,34^ findings from this analysis also confirms the influence of both individual and community factors on the use of 4 ANC visits and institutional delivery. It is important to note that FDC policy interventions might have little influence over some demand barriers such as geographical remoteness, poor transport links, financial barriers in the form of opportunity cost and cost for an onward referral.^3,35^ Thus, tackling all forms of access barriers might be beyond the current scope of FDC policy.

At the individual level, women’s education and household wealth has the strongest positive influence in the utilisation of maternal health services. Higher levels of educational attainment increase the likelihood of attending 4 ANC and utilising institutional delivery. These findings are consistent with previous studies that reported the similar associations.^21,24,25^ One of the common explanations is that education increases the knowledge and awareness on health problems such as when to seek care, where to seek care and how to seek care which in-turn facilitates the ability to process information related to healthy pregnancy behaviors.^21,28^

Several studies demonstrate that household wealth is strongly associated with the use of maternal healthcare services.^21,24,25,28^ It is well argued that the cost associated with care is often the major barrier preventing women from poor households to seek maternal health services.Findings from this study support the common finding that women from higher income households continue to utilise antenatal and delivery services.^21,24,25,28^ This finding may indicate that wealthier women live in better serviced locations and were somehow better informed of available services. Moreover, they may also have the means to pay for same services from a private provider.^24^

Parity was found to have strong negative association with 4 ANC visits and institutional delivery care as observed in similar studies from the developing countries.^21,24,25^ One plausible explanation could be that the perceived risk associated with a first pregnancy is relatively high compared to associated pregnancies. As a result, a woman who have experience of more than one pregnancy and holds the knowledge of delivery care process may not feel the necessity to use maternal health services.^21,24^ Similarly, older women were more likely to attend 4 ANC than younger women. Earlier research suggests that with increasing age there is the increased risk for pregnancy related complication as a result older women were more likely to attend 4 ANC and institutional delivery. In Nepal, young women were generally shy to disclose their pregnancy status as a result they were reluctant to seek care.^24^ These findings are consistent with previous studies that suggested a similar association.^21,25-27^

The effect of caste/ethnicity was present for 4 ANC visits and not for institutional delivery. This was one of a crucial finding suggesting that equal utilisation of institutional delivery services by all caste/ethnicity groups. This finding is supported by earlier evaluations of the Aama programme,^33^ in which it was found that utilisation of institutional delivery services among disadvantaged caste/ethnic groups such as Madhesi and Dalit increased almost two fold since the start of Aama programme.^33^ Nevertheless, some form of reluctance may still persist among the disadvantaged caste/ethnic groups in relation to seeking services that requires multiple visits.

Living in rural locations was generally reported to have strong negative influence on utilisation of maternal health services.^21,24,25^ In this paper, the statement was only found to be true for institutional deliveries and not for 4 ANC visits. Similarly, ecological belt and development region did not influence uptake of 4 ANC visits. One plausible explanation may be the improved accessibility of ANC services through community outreach clinics.^12^ Despite the expansion of birthing centers and strengthening of emergency obstetric care services, institutional deliveries remain more likely for women residing in urban locations, tarai (flat land) and central development region. This disparity in the use of delivery services can be explained by the fact that urban localities have better availability to health facilities as well as better transport links than rural areas. Similarly, the unpredictable nature of onset of labor impedes timely access to health facilities in difficult geographical areas.^25^ The socio-economic composition of the community has been linked with the utilisation of maternal health services by many studies.^21^ This analysis suggests that at the community level, women living in areas with medium to high concentration of rich households were found to have the strongest effect on the use of maternal health services.

## Limitations

Though Anderson model suggests that knowledge does not always lead to utilisation, it could have been interesting to analyse the effect of the policy intervention based on policy treatment ie, differences based on who know or do not know about MIS or Aama program. However, due to data limitation, (NDHS 2006 did not ask questions related to MIS or Aama policies) it could not be done, and thus may pose an important limitation to the extent which causation can be concluded from the association. NDHS data are cross sectional in nature and it limits to draw causal inference with proper reasoning and logic to substantiate the findings. The article provides the analysis against secular time trends in maternal healthcare utilization and may not subscribe causal inference.

## Conclusion

Nepal has made noticeable progress in improving maternal health outcomes. This study suggests that FDC policy has a greater influence in improving the access to and utilisation of 4 ANC and institutional delivery services in Nepal. At the same time, some individual and community level factors continue to hinder improvements in the uptake of maternal health services. It is important to note that uneducated and poorer women living in rural areas face the greatest risk of poor maternal outcomes, including death. A comprehensive policy debate at the national level must be started to ensure equity in service utilisation. Efforts must be channeled to identify barriers and develop strategies that can help underserved women and communities to reap the benefits of FDC policies and programmes. This can be achieved by revising the current FDC policy or identifying combination of policy interventions to produce optimum results.

## Ethical issues

This study utilizes data from Nepal Demographic and Health Survey conducted in different point of time. There were no human subject involved in this study. There are no ethical issues that need to be declared.

## Competing interests

Authors declare that they have no competing interests.

## Authors’ contributions

HB contributed in conceptualizing, desgining, data analysis and writing the manuscript. ST contributed in conceptualizing and writing. TE contributed in quality assurance. DRG supported in data analysis; and TG contributed in conceptualization.

## Authors’ affiliations

^1^Oxford Policy Management/NHSSP, Kathmandu, Nepal. ^2^Oxford Policy Management, Kathmandu, Nepal. ^3^University of Leeds, Leeds, UK. ^4^Volunteer VSO, Nepal.

## Key messages

**Implications for policy makers**

National health policies and programmes are designed to facilitate access to healthcare for all citizen. This paper raises important questions about how to bring poor, uneducated and women living in rural Nepal into the maternal healthcare delivery system. This article suggests the following policy recommendations that would contribute in further improvement of Free Delivery Care (FDC) policies in Nepal:

- This paper concluded that rich, higher caste and urban women benefited more from the current FDC policies and interventions. We recommend Government of Nepal (GoN) to explore the existing barriers among selected groups and revise the FDC policies;
- This paper revealed that women with lower level of education benefited less compared to educated women. We suggest GoN to design and implement specific behaviour change communication strategies for the uneducated women;
- GoN need to ensure the increased utilisation of maternal health services in rural areas. A possible strategy would be strengthening the provision of 24/7 quality delivery services and referral mechanism;
- This paper recognized that 4 ANC users were more likely to utilise institutional delivery services. Thus, we recommend GoN to design a comprehensive incentive package which will cover the pregnancy, delivery and post-delivery conditions; and
- Finally, this paper suggested that FDC policy has greater influence in utilisation of 4 ANC visits and institutional deliveries. However, the influence is skewed towards specific groups. We recommend, GoN to explore the possibilities of designing targeted interventions which will help in reaching the unreached.

**Implications for the public**

This study revealed important findings that can be used to improve utilisation of antenatal and institutional delivery services in Nepal. The data shows that over the study period (1994-2011) the proportion of mothers utilising antenatal and delivery services has significantly increased. This indicates that services offered by the Nepali maternal healthcare delivery system are considered accessible, and acceptable despite a sizeable proportion of pregnant women remain outside of the system. Young, uneducated, poor, indigenous Janjati, Muslims, women from rural areas, and communities with high level of poverty are particularly at risk. More evidence is warranted in order to understand the barriers to utilise maternal healthcare delivery system by these groups.
